# Supplementary material for: TarSynFlow, a workflow for bacterial genome comparisons that revealed genes putatively involved in the probiotic character of Shewanella putrefaciens strain Pdp11
Source: PeerJ. 2019 Feb 28;7:e6526. doi: 10.7717/peerj.6526 (PMC6397758; doi:10.7717/peerj.6526)
Supplement: Supplemental Information 2 — The column order and content is taken just as saved by TarSynFLow. [file peerj-07-6526-s002.pdf]

Supplemental File S2

Table showing the 64 NPS-specific proteins that are absent in the probiotic Pdp11. The result is taken just as saved by TarSynFLow.

UniProt ID	Protein length	Status	Protein description	Gene ontology (GO) terms
A4YAU3	709	unreviewed	Dissimilatory sulfite reductase (EC 1.8.99.-)	periplasmic space [GO:0042597]; copper ion binding [GO:0005507]; heme binding [GO:0020037]; iron ion binding [GO:0005506]; sulfite reductase activity [GO:0016002]; anaerobic respiration [GO:0009061]; hydrogen sulfide biosynthetic process [GO:0070814]
A4YA11	672	unreviewed	Potassium-transporting ATPase ATP-binding subunit	integral component of plasma membrane [GO:0005887]; ATP binding [GO:0005524]; magnesium ion binding [GO:0000287]; potassium-transporting ATPase activity [GO:0008556]
A4YA12	574	unreviewed	Potassium-transporting ATPase potassium-binding subunit	integral component of plasma membrane [GO:0005887]; potassium ion binding [GO:0030955]; potassium-transporting ATPase activity [GO:0008556]
A4YA10	199	reviewed	Potassium-transporting ATPase KdpC subunit	integral component of plasma membrane [GO:0005887]; ATP binding [GO:0005524]; potassium-transporting ATPase activity [GO:0008556]
A4YCJ4	364	unreviewed	Integral membrane sensor signal transduction histidine kinase	integral component of membrane [GO:0016021]; intracellular [GO:0005622]; ATP binding [GO:0005524]; phosphorelay sensor kinase activity [GO:0000155]
A4YAC0	720	unreviewed	TonB-dependent receptor	cell outer membrane [GO:0009279]; receptor activity [GO:0004872]; transport [GO:0006810]
A4YBL1	804	unreviewed	TonB-dependent receptor	cell outer membrane [GO:0009279]; receptor activity [GO:0004872]; transport [GO:0006810]
A4YA08	886	unreviewed	Osmosensitive K+ channel signal transduction histidine kinase	integral component of membrane [GO:0016021]; intracellular [GO:0005622]; ATP binding [GO:0005524]; phosphorelay sensor kinase activity [GO:0000155]

A4YAU6	194	unreviewed	Redoxin domain protein	cell [GO:0005623]; antioxidant activity [GO:0016209]; oxidoreductase activity [GO:0016491]; cell redox homeostasis [GO:0045454]
A4YC21	413	unreviewed	ATP-binding region, ATPase domain protein domain protein	integral component of membrane [GO:0016021]; intracellular [GO:0005622]; ATP binding [GO:0005524]; phosphorelay sensor kinase activity [GO:0000155]
A4YCJ3	242	unreviewed	Two component transcriptional regulator, winged helix family	intracellular [GO:0005622]; DNA binding [GO:0003677]; phosphorelay signal transduction system [GO:0000160]; regulation of transcription, DNA-templated [GO:0006355]; transcription, DNA-templated [GO:0006351]
A4YC22	225	unreviewed	Two component transcriptional regulator, winged helix family	intracellular [GO:0005622]; DNA binding [GO:0003677]; phosphorelay signal transduction system [GO:0000160]; regulation of transcription, DNA-templated [GO:0006355]; transcription, DNA-templated [GO:0006351]
A4Y253	207	unreviewed	Two component transcriptional regulator, LuxR family	intracellular [GO:0005622]; DNA binding [GO:0003677]; phosphorelay signal transduction system [GO:0000160]; regulation of transcription, DNA-templated [GO:0006355]; transcription, DNA-templated [GO:0006351]
A4YAT5	427	unreviewed	Transcriptional regulator, CadC	integral component of membrane [GO:0016021]; intracellular [GO:0005622]; DNA binding [GO:0003677]; phosphorelay signal transduction system [GO:0000160]; regulation of transcription, DNA-templated [GO:0006355]
E6XRR4	506	unreviewed	Flavocytochrome c, flavin subunit (EC 1.3.99.1)	succinate dehydrogenase activity [GO:0000104]
A4Y1H4	607	unreviewed	Glycerophosphoryl diester phosphodiesterase	integral component of membrane [GO:0016021]; glycerophosphodiester phosphodiesterase activity [GO:0008889]; lipid metabolic process [GO:0006629]
A4Y5E5	1185	unreviewed	Uncharacterized protein	integral component of membrane [GO:0016021]; metallopeptidase activity [GO:0008237]; zinc ion binding [GO:0008270]; transport [GO:0006810]

E6XN68	263	unreviewed	Transcriptional regulator, Crp/Fnr family	DNA binding [GO:0003677]; regulation of transcription, DNA-templated [GO:0006355]; transcription, DNA-templated [GO:0006351]
A4Y9T3	487	unreviewed	Transcriptional regulator, GntR family	catalytic activity [GO:0003824]; DNA binding [GO:0003677]; pyridoxal phosphate binding [GO:0030170]; transcription factor activity, sequence-specific DNA binding [GO:0003700]; transcription, DNA-templated [GO:0006351]
A4YC19	582	unreviewed	Flavocytochrome c	membrane [GO:0016020]; FMN binding [GO:0010181]; succinate dehydrogenase activity [GO:0000104]
A4YAW2	239	unreviewed	Transcriptional regulator, GntR family	DNA binding [GO:0003677]; transcription factor activity, sequence-specific DNA binding [GO:0003700]; transcription, DNA-templated [GO:0006351]
A4Y869	545	unreviewed	Phenylalanine/histidine ammonia-lyase	lyase activity [GO:0016829]
E6XRR3	264	unreviewed	Regulatory protein TetR	DNA binding [GO:0003677]; regulation of transcription, DNA-templated [GO:0006355]; transcription, DNA-templated [GO:0006351]
A4Y3C5	334	unreviewed	2OG-Fe(II) oxygenase	iron ion binding [GO:0005506]; oxidoreductase activity [GO:0016491]
A4YBR2	203	unreviewed	Transcriptional regulator, TetR family	DNA binding [GO:0003677]; regulation of transcription, DNA-templated [GO:0006355]; transcription, DNA-templated [GO:0006351]
A4Y4N4	707	unreviewed	Aldehyde ferredoxin oxidoreductase (EC 1.2.7.5)	aldehyde ferredoxin oxidoreductase activity [GO:0033726]; electron carrier activity [GO:0009055]; iron-sulfur cluster binding [GO:0051536]
A4Y4N9	296	unreviewed	Uncharacterized protein	
A4YAT9	163	unreviewed	NosL family protein	
A4Y4N3	214	unreviewed	4Fe-4S ferredoxin, iron-sulfur binding domain protein	
A4YAU2	146	unreviewed	Rhodanese domain protein	

A4YBE8	183	unreviewed	Uncharacterized protein	
A4Y3R6	161	unreviewed	Uncharacterized protein	
A4Y8G6	184	unreviewed	Sugar transferase	integral component of membrane [GO:0016021]; transferase activity [GO:0016740]
A4YC20	254	unreviewed	Uncharacterized protein	cell outer membrane [GO:0009279]
A4YAW6	116	unreviewed	Uncharacterized protein	
A4Y4V6	156	unreviewed	Uncharacterized protein	
A4Y600	384	unreviewed	Major facilitator superfamily MFS_1	integral component of membrane [GO:0016021]; transmembrane transport [GO:0055085]
A4Y3K8	342	unreviewed	Outer membrane porin, putative	
A4Y3S5	273	unreviewed	Cytochrome c, putative	
A4YAD3	176	unreviewed	Uncharacterized protein	
A4YBR1	239	unreviewed	ThiJ/PfpI domain protein	
A4Y847	143	unreviewed	Uncharacterized protein	
A4YAU0	226	unreviewed	Formate-dependent nitrite reductase, NrfG protein	integral component of membrane [GO:0016021]
A4Y5E4	292	unreviewed	ABC transporter related	ATP binding [GO:0005524]; ATPase activity [GO:0016887]
A4Y5F4	171	unreviewed	Uncharacterized protein	
A4Y601	226	unreviewed	Cyclic nucleotide-binding protein	
A4YAB5	201	unreviewed	Uncharacterized protein	
A4Y6D0	103	unreviewed	Transport-associated	
A4YA09	256	unreviewed	Uncharacterized protein	
A4YBE9	150	unreviewed	Uncharacterized protein	
A4Y3K9	298	unreviewed	Transcriptional regulator, LysR family	DNA binding [GO:0003677]; transcription factor activity, sequence-specific DNA binding [GO:0003700]; transcription, DNA-templated [GO:0006351]

A4YCB6	352	unreviewed	CDP-glycerol:poly(Glycerophosphate) glycerophosphotransferase	membrane [GO:0016020]; CDP-glycerol glycerophosphotransferase activity [GO:0047355]
A4Y6G5	96	unreviewed	Cytochrome c3	
A4YAW5	557	unreviewed	Fumarate reductase/succinate dehydrogenase flavoprotein domain protein	oxidoreductase activity [GO:0016491]
A4Y3V6	365	unreviewed	Sodium/calcium exchanger membrane region	integral component of membrane [GO:0016021]; transmembrane transport [GO:0055085]
A4Y5W9	130	unreviewed	Uncharacterized protein	
A4YAT8	431	unreviewed	Periplasmic copper-binding	
A4YAU5	139	unreviewed	Cytochrome C biogenesis protein	integral component of membrane [GO:0016021]
E6XRR5	123	unreviewed	Flavocytochrome c, heme submit	
E6XFL2	22	unreviewed	LeuABCD operon attenuation leader peptide, LeuL	
E6XRR8	246	unreviewed	Putative uncharacterized protein	
E6XLJ3	146	unreviewed	Putative uncharacterized protein	
E6XJB6	156	unreviewed	Putative uncharacterized protein	
E6XRR6	377	unreviewed	Outer membrane beta barrel protein	
