# Supplementary material for: TarSynFlow, a workflow for bacterial genome comparisons that revealed genes putatively involved in the probiotic character of Shewanella putrefaciens strain Pdp11
Source: PeerJ. 2019 Feb 28;7:e6526. doi: 10.7717/peerj.6526 (PMC6397758; doi:10.7717/peerj.6526)

Supplemental File S3

Similarity between sequence amplicons and Pdp11 genome sequence. The table contains the tabulated blast output when amplicon sequences were queried using the Ppd11 genome as subject database. Then, QV plots for the two amplicon sequences whose identity was lower than 96% (A4Y1U2 and E6XLE5) was shown to demonstrate that sequence divergence is explained by low QV values rather than real sequence divergences. The QV plot for Q8GJK1 is also included as reference of amplicon sequence with 'good' quality.

Amplicon sequence file as query	Subject ID	% identity	Align. length	Mismatch count	Gap open count	Query start	Query end	Subject start	Subject end	e-Value	Score
170201-063_C17_G14_Pdp11_E6XG14_R.ab1	Sp_Pdp11	96.73	1222	21	18	6	1209	3863413	3864633	0.0	2017
170125-078_E11_G15_Pdp11_E6XG15_F.ab1	Sp_Pdp11	98.25	571	9	1	13	582	3865845	3865275	0.0	998
170125-078_E13_LE5_Pdp11_E6XLE5_F.ab1	Sp_Pdp11	88.25	366	39	4	136	501	3454748	3455109	3e-122	435
170201-063_A03_k1_Pdp11_Q8GJK1_R.ab1	Sp_Pdp11	99.30	859	2	4	10	866	4298959	4298103	0.0	1550
170201-063_G03_R2_Pdp11_Q6ZYR2_F.ab1	Sp_Pdp11	99.71	1023	2	1	20	1041	4390569	4391591	0.0	1871
170201-063_C01_U2_Pdp11_A4Y1U2_F.ab1	Sp_Pdp11	78.41	602	117	13	12	610	3192525	3191934	3e-105	379

QV plot for A4Y1U2

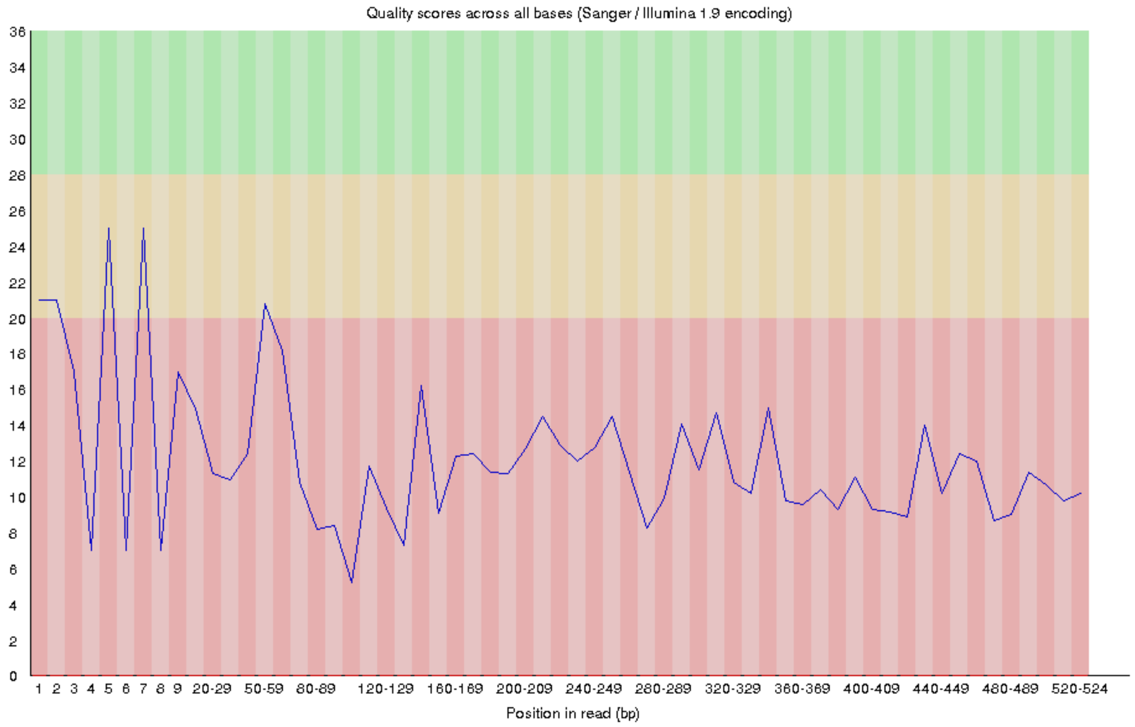

QV plot for E6XLE5

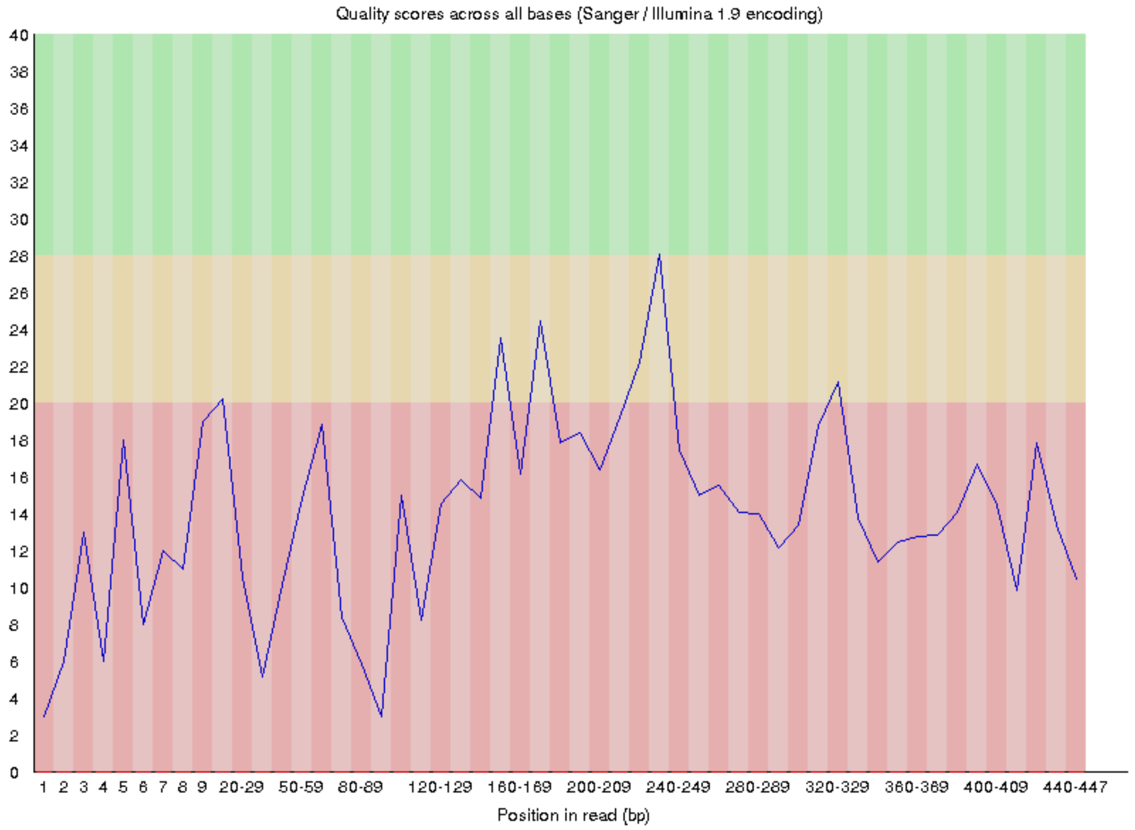

QV plot for Q8GJK1

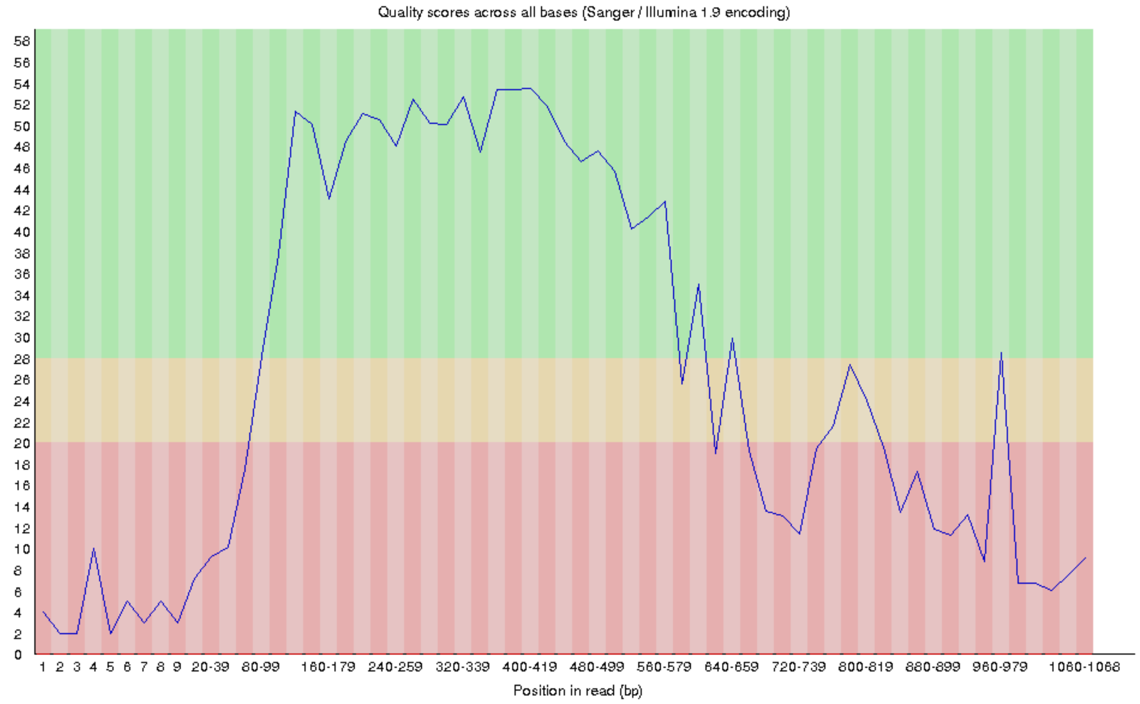

Supplement: Supplemental Information 3 — The table contains the tabulated blast output when amplicon sequences were queried using the Ppd11 genome as subject database. Then, QV plots for the two amplicon sequences whose identity was lower than 96% (A4Y1U2 and E6XLE5) was shown to demonstrate that sequence divergence is explained by low QV values rather than real sequence divergences. The QV plot for Q8GJK1 is also included as reference of amplicon sequence with ‘good’ quality. [file peerj-07-6526-s003.pdf]
